# Development and characterization of sphingosine 1-phosphate receptor 1 monoclonal antibody suitable for cell imaging and biochemical studies of endogenous receptors

**DOI:** 10.1371/journal.pone.0213203

**Published:** 2019-03-07

**Authors:** Franck Talmont, Lionel Moulédous, Marion Baranger, Anne Gomez-Brouchet, Jean-Marie Zajac, Clarence Deffaud, Olivier Cuvillier, Anastassia Hatzoglou

**Affiliations:** 1 Institut de Pharmacologie et de Biologie Structurale, Université de Toulouse, CNRS, UPS, Toulouse, France; 2 BIOTEM, Apprieu, France; 3 Service d’anatomie et cytologie pathologiques, IUCT Oncopole, Toulouse, France; Instituto Butantan, BRAZIL

## Abstract

Although sphingosine-1-phosphate receptor 1 (S1P_1_) has been shown to trigger several S1P targeted functions such as immune cell trafficking, cell proliferation, migration, or angiogenesis, tools that allow the accurate detection of endogenous S1P_1_ localization and trafficking remain to be obtained and validated. In this study, we developed and characterized a novel monoclonal S1P_1_ antibody. Mice were immunized with S1P_1_ produced in the yeast *Pichia pastoris* and nine hybridoma clones producing monoclonal antibodies were created. Using different technical approaches including Western blot, immunoprecipitation and immunocytochemistry, we show that a selected clone, hereinafter referred to as 2B9, recognizes human and mouse S1P_1_ in various cell lineages. The interaction between 2B9 and S1P_1_ is specific over receptor subtypes, as the antibody does not binds to S1P_2_ or S1P_5_ receptors. Using cell-imaging methods, we demonstrate that 2B9 binds to an epitope located at the intracellular domain of S1P_1_; reveals cytosolic and membrane localization of the endogenous S1P_1_; and receptor internalization upon S1P or FTY720-P stimulation. Finally, loss of 2B9 signal upon knockdown of endogenous S1P_1_ by specific small interference RNAs further confirms its specificity. 2B9 was also able to detect S1P_1_ in human kidney and spinal cord tissue by immunohistochemistry. Altogether, our results suggest that 2B9 could be a useful tool to detect, quantify or localize low amounts of endogenous S1P_1_ in various physiological and pathological processes.

## Introduction

Sphingosine 1-phosphate receptor 1 (S1P_1_) is part of the sphingosine 1-phosphate (S1P) receptor family, which comprises five G-protein coupled receptors (GPCR, S1P_1_, S1P_2_, S1P_3_, S1P_4_, and S1P_5_, S1P_1-5_). This receptor family, firstly named, endothelial differentiation gene (EDG) family of lipid receptors, also comprises lysophosphatidic acid (LPA) receptors. S1P_1-5_ bind the switterionic lysophospholipid S1P, with low nanomolar affinities, share sequence, and genomic structure similarities [1–3]. S1P_1_ was originally detected in human umbilical vein endothelial cells (HUVEC) treated by phorbol 12-myristate 13-acetate [4]. S1P_1_ signaling pathway includes coupling to the Gi/o proteins family and hence inhibition of adenylyl cyclase, activation of phosphatidylinositide 3-kinase and phospholipase C [5]. Analysis of transcripts indicates that S1P_1_ is strongly expressed in adipose tissues, spleen, lung, brain, liver, and heart and poorly represented in skeletal muscle, thymus, uterus, and kidney of adult mice [6]. When S1PR1 gene was ablated in the germ line of mice it resulted in a lethal effect *in utero* [7]. In fact S1P_1_ has a vital role in vascular development and lethality in mice was due to a defect in blood vessels development [6]. S1P_1_ has also an essential function in cell migration, in particular in the drain of T cells from the thymus to the blood and surrounding lymphoid structures [8]. More particularly, the activation of S1P_1_ signaling pathway with an agonist prevents the recruitment and migration of lymphocytes to sites of inflammation by the loss of ability to perceive S1P gradient concentration. The drug FTY720 (Fingolimod, Gilenya) which activates S1P_1_ leading to impaired lymphocyte migration is currently used for the treatment of relapsing remitting multiple sclerosis [9]. This drug is phosphorylated, *in vivo*, and the resulting FTY720-P binds to S1P_1_ to activate receptors as a true agonist. Nevertheless, this process leads to the internalization of S1P_1_ that are not recycled at the membrane thus blocking the egress of lymphocytes. S1P_1_ is also implicated in cancer-related processes such as neovascularization in a tumor microenvironment context, cell migration, survival, transformation and progression [10]. Thus, the development of accurate tools for the detection, quantitation and localization of S1P_1_ is mandatory to understand the implication of this receptor in the regulation of numerous physiological and pathological processes. Besides commercial antibodies used by research groups, which are mainly rabbit polyclonal, generated with peptidic antigens and badly characterized, the analysis of scientific literature on S1P_1_ allows selecting anti-S1P_1_ antibodies demonstrating rather good efficacy. The murine anti-S1P1 monoclonal IgG, called E49 [11] was produced using an *Escherichia coli*-derived human S1P_1_ full-length antigen. Another interesting antibody was the rabbit anti-S1P_1_ polyclonal antibody H60 raised against amino acids 322–381 of S1P_1_ of human origin [9, 12, 13]. Unfortunately, all these antibodies were discontinued. In this context, we have generated a murine monoclonal anti-S1P_1_ antibody using a purified protein produced in the methylotrophic yeast *Pichia pastoris* model [14]. Mice were immunized with purified S1P_1_ and nine hybridoma clones secreting specific S1P_1_ monoclonal antibodies (MAbs) were produced. Among these, 2B9 was selected and further characterized. This antibody specifically recognizes human recombinant cmyc-S1P_1_ and S1P_1_-Green Fluorescent Protein, as well as human and mouse native S1P_1_s. We provide evidence that 2B9 recognizes endogenous S1P_1_ in murine embryonic fibroblasts (MEF), BT-549 breast cancer cell line and HUVEC cells. The binding of 2B9 to S1P_1_ is specific since the knocking down of the receptor in cells leads to the loss of signal. Furthermore, 2B9 was able to detect S1P_1_ by immunohistochemistry in human tissue. Finally, 2B9 binds to the intracellular part of the receptor, reveals cytoplasmic and membrane bound S1P_1_ as well as receptor internalization upon S1P and FTY720-P stimulation.

## Methods

### Plasmid construction

Plasmid cmyc-tagged pcDNA3-S1P_1_ (Dr James Van Brockyn’s gift) was modified by PCR (polymerase chain reaction) at the 5’ end to introduce a BstBI enzyme restriction site and at the 3’ end to introduce a Xba I site. Oligonucleotides were 5’-TTATTCGAAACGATGGGGCC CACCAGCGTC-3’ (BstBI forward) and 5’-TTGTTCTAGAGGGGAAGAAGAGTTGA CGTT-3’ (XbaI reverse). Modified cDNA was introduced into a TOPO TA vector (Invitrogen, Carslbad, CA). After digestion with BstBI and Xba enzymes, cDNA was introduced into pPICZ-hMOR-cmyc-his [15] vector digested with BstBI and XbaI thus deleting the hMOR coding sequence and leading to pPICZ-hS1P_1_-cmyc-his vector. This vector contains the full length S1P_1_R gene in fusion with cmyc and 6-histidine tags. Mouse full-length S1P_1_/EDG1 versaclone cDNA (RD systems) was cloned in pcDNA3 (Invitrogen, Carslbad, CA) using BamHI and XbaI restriction enzymes to obtain mS1P_1_-pcDNA3 plasmid.

### Preparation of immunogens

S1P_1_ was expressed in *Pichia* cells by electro-transformation and cells were plated on zeocin 100μg/ml containing solid medium. Ten clones were selected and grown for 48h at 30°C in two ml of a glycerol containing liquid medium (BMGY). S1P_1_ expression was induced in 2 ml liquid medium containing 1% methanol after centrifugation of cells and discarding of glycerol medium. Cells were then centrifuged and broken with glass beads. After elimination of particulate matter and unbroken cells at 1,000g, a fraction, for each clone, was prepared by centrifugation at 10,000g and analyzed by WB using anti-cmyc antibodies [14, 15].

### Immunization of mice

BIOTEM animal experiments were realized in accordance with the dedicated laws. Institutional Animal Care Committee (IACUC) was DDPP de l’Isère. BIOTEM Ethics committee was the approving committee. The pure full-length receptor in complete Freund’s adjuvant was injected (100 μg) subcutaneously in OF1 mice. Mice were subsequently immunized two times with hS1P_1_ (100 μg) in incomplete Freund’s adjuvant. Pure receptors (200 μg) were administrated intraperitoneally in mice three days before cell fusion between splenocytes and myeloma cell line (NS-1). The selection of secreting Hybridomas was performed in Hypoxanthine-Aminopterin-Thymidine medium. Before being euthanized using carbon dioxide method, mice were bled to collect immune serum.

### Cell culture and transfections

CHO cells expressing human sphingosine 1-phosphate receptor 1 fused to the green fluorescent protein (CHO hS1P_1_-GFP; Dr Kevin R. Lynch’ gift) and CHO wild type (WT) cells were grown as described [16]. Mouse embryonic fibroblasts (MEF) and HUVEC cells were used until passage seven and cultured as previously described [17]. Human breast cancer cells (BT-549) and human Embryonic Kidney 293 cells (HEK) were from ATCC (Manassas, VA, USA). Cells were cultured in their optimal conditions in DMEM (Gibco, Grand Island, NY), containing 10% fetal bovine serum (FBS, Gibco), 100 U/mL penicillin (Sigma, St. Louis, MO), and 100 mg/mL streptomycin (Sigma, St. Louis, MO) at 37 °C in a humidified incubator with 5% CO_2_ atmosphere. To establish cell lines stably expressing human S1P_1_, mouse S1P_1_ (cDNA from RD systems, a bio-techne brand) and GFP [18], CHO WT cells were transfected with the appropriate expression plasmids using Lipofectamine 2000 (Life Technologies) according to the manufacturer’s instructions. 48 hours post transfection, CHO cells expressing human, mouse S1P_1_ and control empty pcDNA3 plasmid were selected by adding 400 μg/ml G418 (Euromedex) whereas 500 μg/ml Hygromycin (Gibco) was added to the culture medium for the selection of GFP-CHO cells. Selected clones were analyzed by Western blot and immunocytochemistry. Transient expression of cmyc-S1P_1_, cmyc-S1P_2_ and cmyc-S1P_5_ (Dr James Van Brockyn’s gift) was performed in Human Embryonic Kidney 293 cells (HEK) using Lipofectamine 2000. SiRNA transfections were performed using Lipofectamine 2000 (Invitrogen) according to manufacturer instructions with a mix of two siRNAs (siS1P_1_A: sens 5’ GAGUUAGUUCCUGUGAACAdTdT 3’ and antisens 5’ UGUUCACAGGAACUAACUCdTdT 3’ and siS1P_1_B: sens 5’ CUGACUACGUCAACUAUGAdTdT 3’ and antisens 5’ UCAUAAGUUGACGUAGUCAGdTdT 3’) as previously described [19]. SiRNAs negative control (siScr) was from Eurogentech.

### Immunoprecipitation and Western blot analysis

After culture, cells recovered by scratching were centrifuged for 15 min at 1,000g and maintained for at least 24 hours at—80°C. Cells were lysed with a Potter Elvehjem homogenizer and cell membranes were obtained after two consecutive centrifugations at 1,000g and 100,000g. For immunoprecipitation, cell membranes were solubilized by incubation in a lysis buffer containing 0.5% NP40 (Calbiochem) and protease inhibitor cocktail (Roche Applied Science, France) at 4°C for 1 night [20]. Samples were centrifuged at 100,000g and the supernatant was precleared by contact with Protein G sepharose (GE Healthcare Life Science) for 1h at 4°C. The supernatant was then incubated with 2B9 and Protein G sepharose for 20 hours. Immunoprecipitated samples were washed with lysis buffer and solubilized in a Laemmli buffer, denatured for five min at 100 °C before being analyzed by SDS-PAGE and Western blot using 1:2,000 anti-GFP rabbit polyclonal antibody (sc-8334, Santa Cruz) or 1:1,000 anti-S1P_1_ H60 rabbit polyclonal antibody (sc-25489, Santa Cruz). S1P_1_ containing samples from *Pichia pastoris*, CHO or HEK cell membranes or pure S1P_1_ were analyzed by SDS-PAGE by Western-blot or dot blot as previously described using 1:1,000 anti-cmyc mouse monoclonal antibody (clone 2E10, Sigma) [14].

### Immunocytochemistry

Immunofluorescence studies were conducted as previously described [21, 22]. Briefly cells were plated on glass coverslips and treated as indicated in the figure legends. Cells were washed with an ice-cold phosphate-buffered saline (PBS), fixed in 4% (w/v) paraformaldehyde (Electron Microscopy Science, PA) for 10 min at room temperature. Cells were permeabilized with 0.2% Triton X-100 in PBS for 7 min 5% of fetal calf serum in PBS was used to block non specific sites during 45 min Cells were then incubated with primary antibodies (2B9; H60; IgG2A, 1 mg/ml, dilution 1:75 w/v) for 45 min followed by secondary antibodies conjugated to the relevant fluorochrome (Alexa 488-coupled antibodies or TR-coupled antibodies from Molecular Probes, dilution 1:1500 v/v) for 45 min DNA was stained with DAPI (4′,6-diamidino-2-phenylindole). To identify the S1P_1_ binding domain, non-permeabilized or Triton X-100-permeabilized cells were stained as above. For receptor internalization studies, cells were seeded on glass coverslips and 24 hours later cells were serum-starved during 2 hours prior treatment. Cells were treated with vehicle (Ctrl) or S1P (1μM) or FTY720-P (1μM) for 1 hour. Then, cells were washed twice with PBS, fixed and stained with 2B9 and DAPI as described above. Image acquisitions were performed on a Nickon-Eclipse Cil; DS-Q12, microscope using 40x or 60x oil immersion objective and image analysis was conducted with ImageJ software.

### Immunohistochemistry

Frozen human spinal cord sections from BioChain Institute (Newark, USA) were post-fixed in 4% formaldehyde for 15 min at room temperature. Sections were washed twice with PBS, and then incubated with 0.3% H_2_O_2_ in PBS for 30 min. After two additional washes, blocking was performed for 2 hours in PBS containing 0.25% Triton X-100 (PBS-T) and 3% normal goat serum (Vector Laboratories, Burlingame, CA, USA). Sections were then incubated with antibodies diluted 1:4,000 in the same buffer for 48 hours at 4°C. After three 10 min washes with PBS-T, sections were incubated with biotinylated goat anti-mouse antibody (Vector Laboratories) diluted 1:600 in blocking solution for 2 hours at room temperature. After three 10 min washes, sections were placed in horseradish peroxidase avidin-biotin complex (Vectastain ABC kit, Vector Laboratories) diluted in PBS-T for 1 hour at room temperature. Sections were finally rinsed in PBS and stained in 3,3’-diaminobenzidine substrate kit (Vectastain DAB kit, Vector Laboratories) for 8 min according to manufacturer instructions. Sections were rinsed, dehydrated in graded ethanol, cleared in toluene and placed under coverslips with Deppex. Sections were viewed under a Leica CTR 600 wide-field microscope (Nanterre, France) and the Mercator software (Explora Nova, La Rochelle, France) was used to take pictures with 5x objective.

Human kidney samples were provided by the Centre de Ressources Biologiques-Cancer (CRB-Cancer, Institut Universitaire du Cancer Toulouse-Oncopole). Immunostaing using 2B9 and control IgG2A antibodies (1:50) were performed on kidney tissue microarrays (TMA) as previously described [23].

## Results

### Production of pure receptors

**Fig 1:**
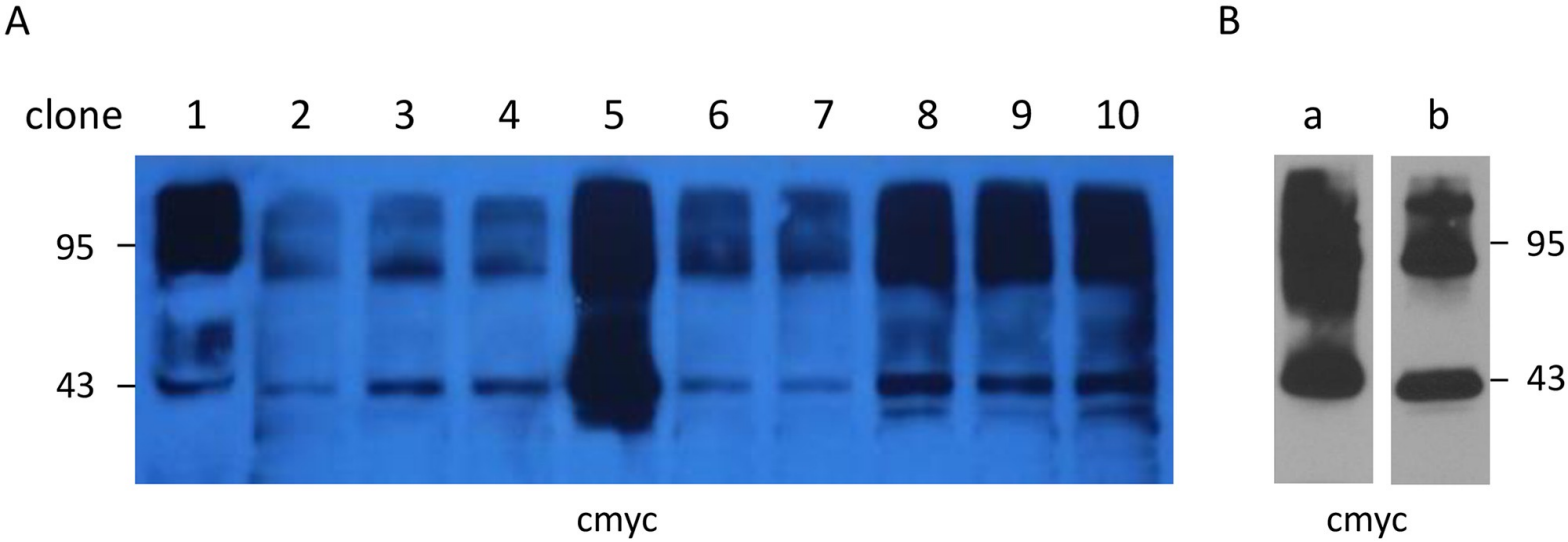
Selection of *Pichia pastoris* clones expressing hS1P_1_ and receptor purification. **A** Expression of hS1P_1_ in different transfected *Pichia pastoris* clones (1 to 10). **B** (**a**) Purified hS1P_1_ eluted with 300 mM imidazole. (**b**) Expression of hS1P_1_ in crude supernatant from *Pichia pastoris* after cell breakage and centrifugation at 1,000g. Detection of receptors were realized after SDS-PAGE and Western blotting using an anti-cmyc antibody (1:1,000, v/v).

All *Pichia* clones presented the same pattern, specifically a protein monomeric band at 43 kDa and multimeric protein bands over 95 kDa (Fig 1A). The best expressing clone (clone 5) was chosen for overexpression in shacked flasks. A total of 43 g of wet cells were broken with glass beads and used to prepare a fraction centrifuged at 10,000g. After solubilization in 0.1% sodium dodecyl sulfate, interaction with the Nickel phase and elution with imidazole, 5 mg of pure receptors were produced (Fig 1Ba) presenting the same Western blot pattern as observed in *Pichia* cell membranes (Fig 1Bb).

### Nine anti-S1P_1_ antibodies for one thousand clones tested

**Fig 2:**
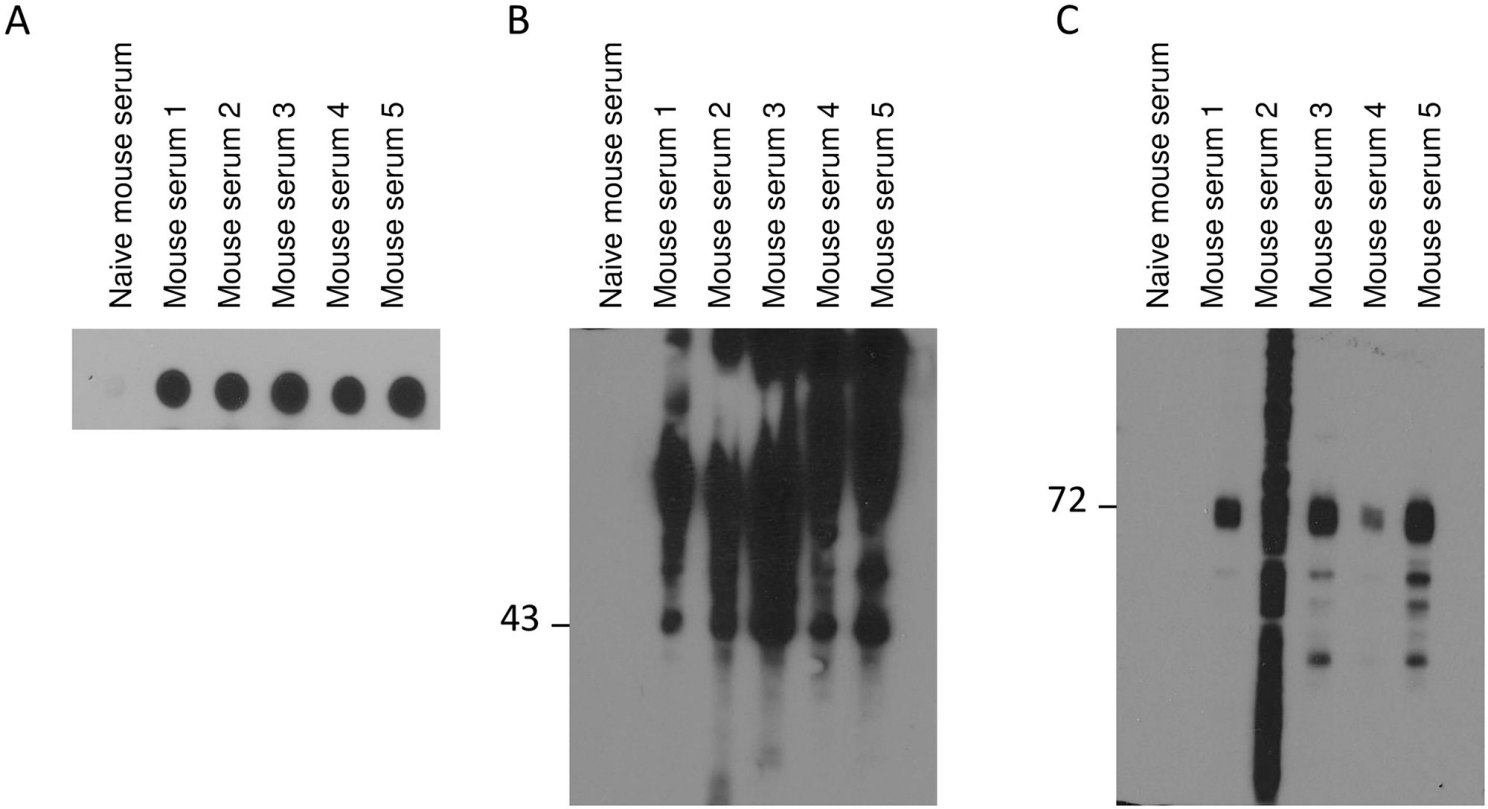
Assessment of mouse sera. **A** Dot blot of CHO hS1P_1_-GFP cell membranes (1.6 μg of sample was spotted onto the PVDF (polyvinylidene fluoride) membrane and revealed with 1:1,000 v/v diluted sera). **B** Pure hS1P_1_-cmyc from *Pichia* yeast cells (1 μg of sample per lane) was detected by Western blot using sera from five different immunized mice (1:5,000 v/v). **C** hS1P1 was detected in membrane fractions from CHO expressing hS1P_1_-GFP cells (20 μg of sample per lane) using sera from five different immunized mice (1:5,000 v/v).

Pure human S1P_1_ antigens from *Pichia pastoris* were used to immunize mice and to generate antibodies specific to receptors. Five animals were immunized with the antigen solubilized in 0.1% SDS (mouse 1–5). To select animals optimally responding to the recombinant proteins, reactivity against S1P_1_ was assessed by dot blot and Western blot with sera obtained from the animals (Fig 2). All sera from immunized mice detected S1P_1_ from stable CHO cell line expressing S1P_1_-GFP by dot blot (Fig 2A). All sera were able to recognize pure S1P_1_-cmyc from *Pichia* yeast (Fig 2B) and four out of five sera detected specifically S1P_1_-GFP (Fig 2C) by Western blot. Serum from naïve mouse was unable to detect receptors either by dot or Western blot. Mice 3 and 4 were selected and a fusion was performed between spleen cells and myeloma NS-1 cell line by using standard procedures. From one thousand hybridoma clones screened as above, nine produced S1P_1_ specific antibodies that were further characterized. Here we present results of one antibody called 2B9. This clone was chosen because it exhibited, by Western blot, the more intense and clean signal among all clone tested on S1P_1_-GFP membranes. Not all clones presented the same productivity and 2B9 was one of the best secreting clones.

### Specificity of 2B9 was evaluated by Western blot and immunoprecipitation

**Fig 3:**
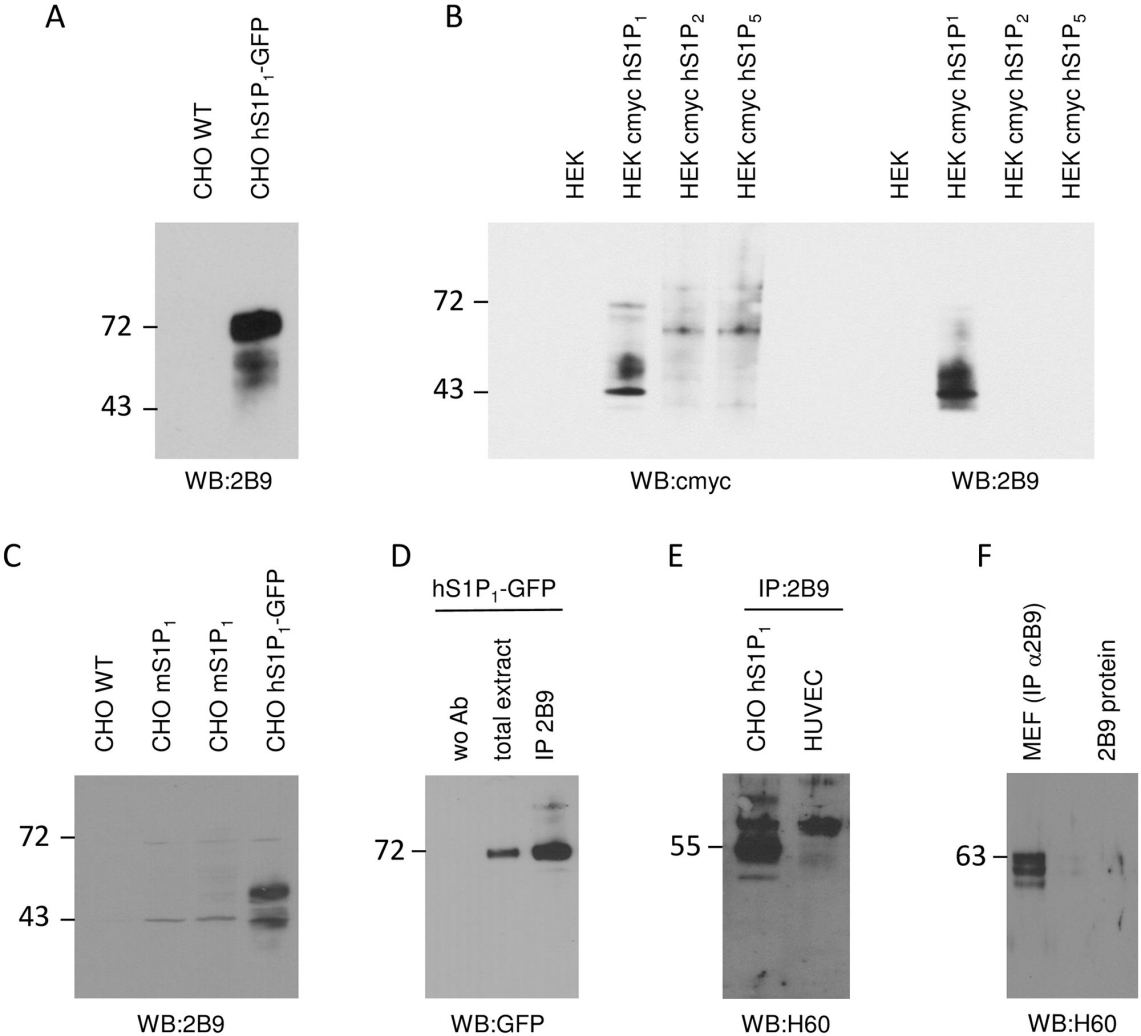
Detection of receptors in membranes from recombinant CHO and HEK cells by Western blot and in HUVEC and MEF after immunoprecipitation. **A** Expression of hS1P_1_ in control CHO WT cells and in CHO cells stably expressing hS1P_1_-GFP using 2B9 antibodies (1:5,000 w/v dilution). **B** HEK cells were transfected with vector expressing cmyc-hS1P_1_, cmyc-hS1P_2_ or cmyc-hS1P_5_. Expression of the receptors was detected 48 hours later using cmyc (1:1,000 v/v) and 2B9 (1:5,000 w/v) antibodies. **C** Detection of mouse S1P_1_ in control CHO WT cells, CHO cells stably expressing mS1P_1_ and CHO cells stably expressing hS1P_1_-GFP using 2B9 antibody (1:1,000 w/v). **D** Human S1P_1_ was immunoprecipitated from CHO cells stably expressing hS1P_1_-GFP using 2B9 antibody and detected with rabbit anti-GFP antibodies (1:2,000, v/v). Negative control was realized with no antibodies and positive control with a detergent-solubilized extract. **E** Endogenous human S1P_1_ was immunoprecipitated from HUVEC cells using 2B9 antibody and detected with rabbit anti-S1P_1_ H60 antibodies (1:1,000, v/v). CHO hS1P_1_ were used as positive control. **F** Endogenous mS1P_1_ was immunoprecipitated from MEF membrane fraction using 2B9 antibodies and detected with rabbit anti-S1P_1_ H60 antibodies (1:1,000, v/v). 2B9 proteins were used as a control.

We first tested the capacity of 2B9 to detect S1P_1_ protein by Western blot. As shown in Fig 3A, 2B9 detected S1P_1_ in extracts from CHO cells stably expressing hS1P_1_-GFP but not from wild type CHO cells that do not express any S1P receptor as formerly described [24]. In HEK cells transiently expressing recombinant human cmyc-hS1P_1_, cmyc-hS1P_2_ or cmyc-hS1P_5_, cmyc antibody detected all receptors whereas 2B9 bound to hS1P_1_ but not hS1P_2_ and hS1P_5_ further confirming its specificity (Fig 3B). 2B9 also recognized mouse S1P_1_ and human S1P_1_-GFP overexpressed in CHO cells (Fig 3C) and was able to immunoprecipitate the recombinant human S1P_1_-GFP as revealed with anti-GFP antibody (Fig 3D). Intensity of Western blot mouse S1P_1_ detection was low and this is a weakness for direct detection of mS1P_1_ by this technic. Untagged hS1P_1_ (Fig 3E) stably expressed in CHO cells was also immunoprecipitated by 2B9 and detected with rabbit H60 anti-S1P_1_ antibody, [11, 25]. Furthermore, 2B9 precipitated endogenous human S1P_1_ from HUVECs (Fig 3E) that were previously shown to express S1P_1_ receptors [9, 26]. Lastly, 2B9 was also able to precipitate endogenous mouse S1P_1_ in MEF while purified antibody protein 2B9 was used as control (Fig 3F).

### 2B9 detects endogenous S1P_1_ by immunofluorescence microscopy

**Fig 4:**
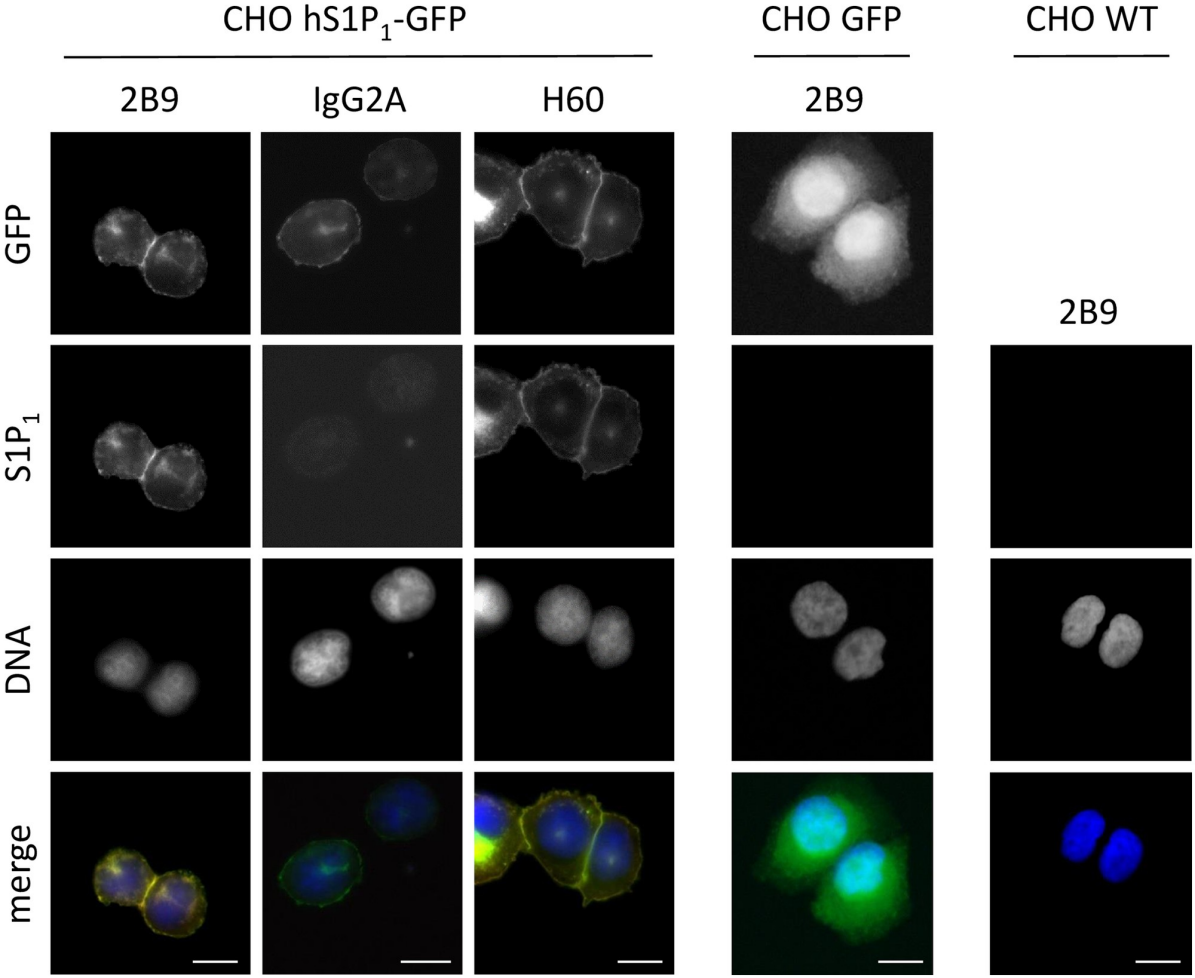
2B9 specifically recognizes recombinant S1P_1_. CHO cells stably expressing hS1P_1_ (CHO hS1P_1_-GFP) or GFP (CHO GFP) and CHO WT cell lines were seeded on coverslips and 24h later were fixed and stained for S1P_1_ (red) with 2B9 or H60 or control IgG2A antibodies (1:100, w/v). Cells were treated with DAPI to visualize nuclei (blue). Colocalization (yellow) is shown between GFP (green) and 2B9 (red). Images are representative of the population examined. Scale bar, 10 μm.

**Fig 5:**
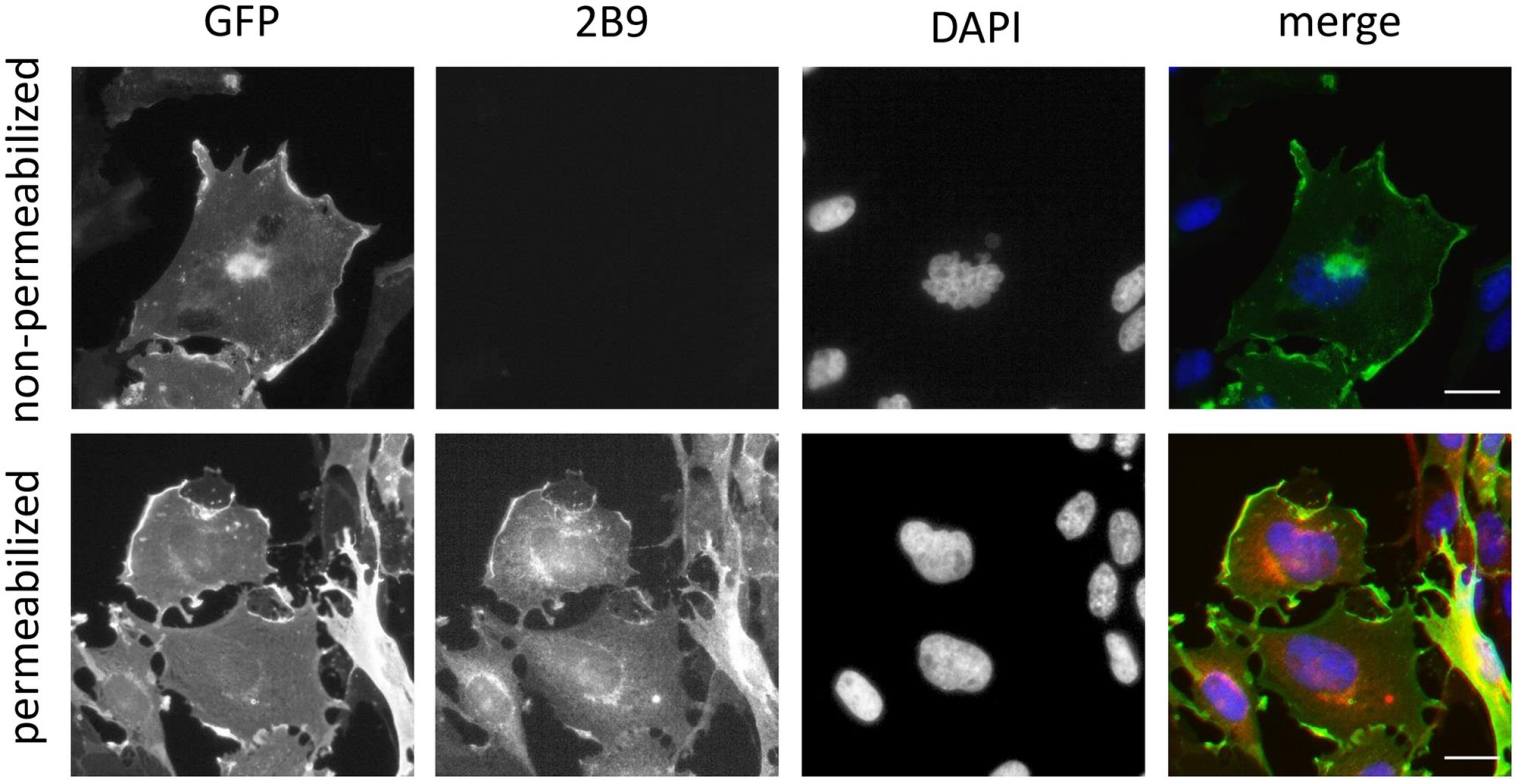
2B9 binds to the intracellular domain of S1P_1_. CHO S1P_1_-GFP cells were seeded on coverslips and fixed 24h later. Permeabilized and non-permeabilized cells were stained for S1P_1_ with 2B9 (red) and for DNA with DAPI (blue). Colocalization (yellow) is shown between GFP (green) and 2B9 (1:100, w/v, red) in permeabilized cells. Non-permeabilized cells are not stained with 2B9 (1:100, w/v, red). Images are representative of the population examined. Scale bar, 10 μm.

**Fig 6:**
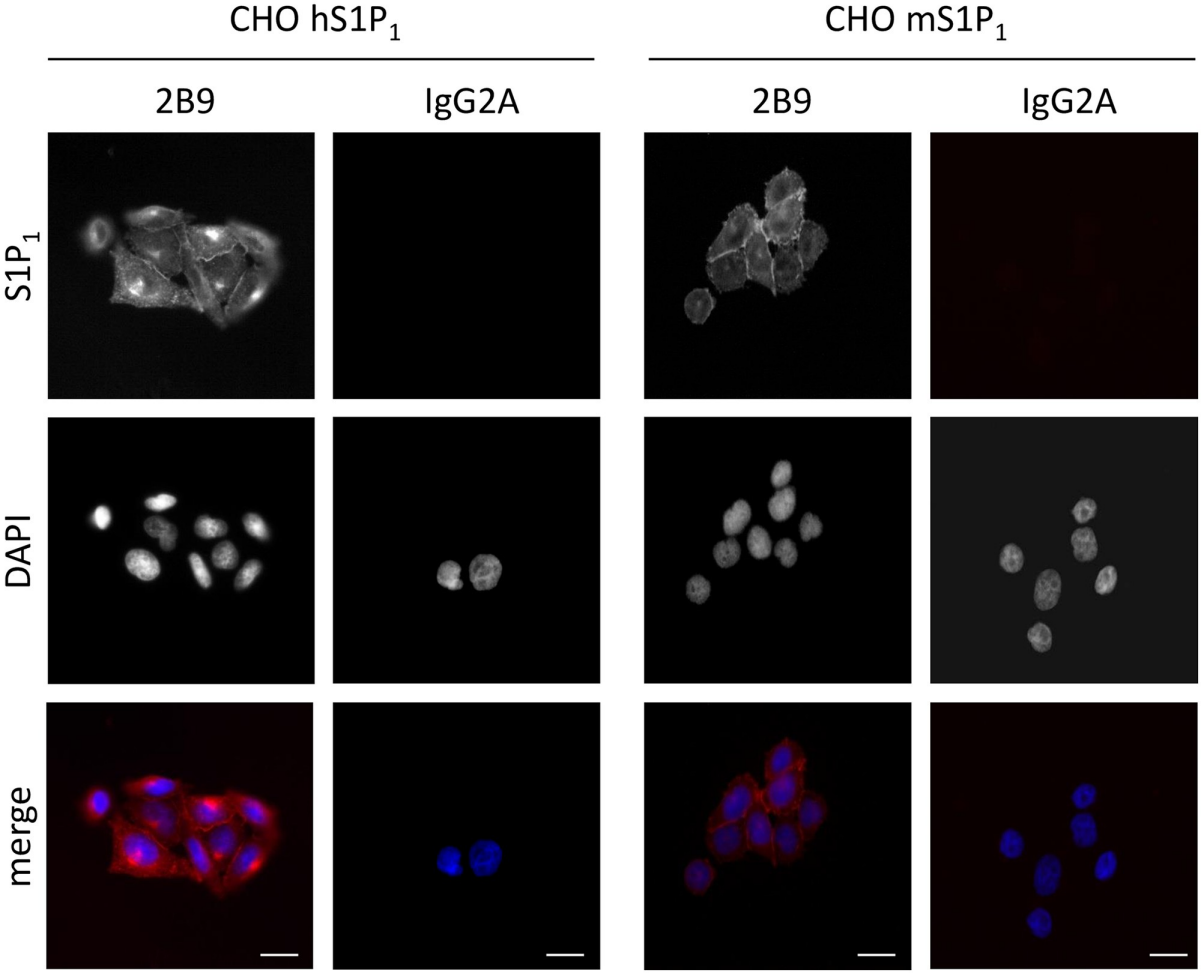
2B9 recognizes human and mouse S1P_1_. CHO cells stably expressing human (CHO hS1P_1_) or mouse (CHO mS1P_1_) S1P_1_ receptors were fixed and stained with 2B9 (red) or control IgG2A (red) antibodies (1:100, w/v). DNA was stained with DAPI (blue). Images are representative of the population examined. Scale bar, 10 μm.

**Fig 7:**
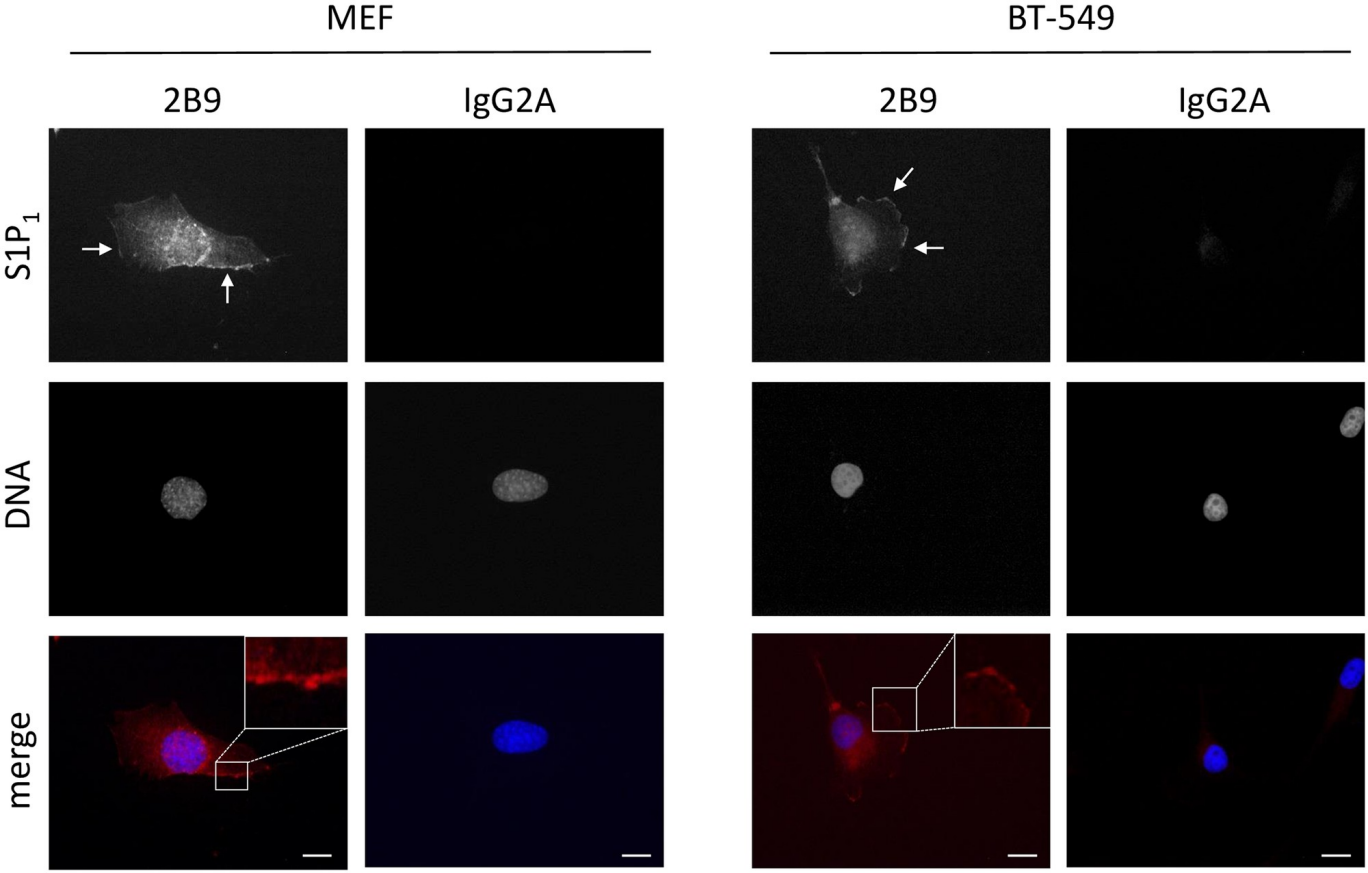
2B9 recognizes endogenous S1P_1_. MEF and BT-549 cells were fixed and stained with 2B9 (1:100, w/v, red) or IgG2A (1:100, w/v, red) antibodies and DAPI (blue). Plasma membrane and cytoplasmic localization is seen with 2B9. Arrows indicate S1P_1_ staining at the level of the plasma membrane. Inserts represent magnified regions. Images are representative of the population examined. Scale bar, 10 μm.

**Fig 8:**
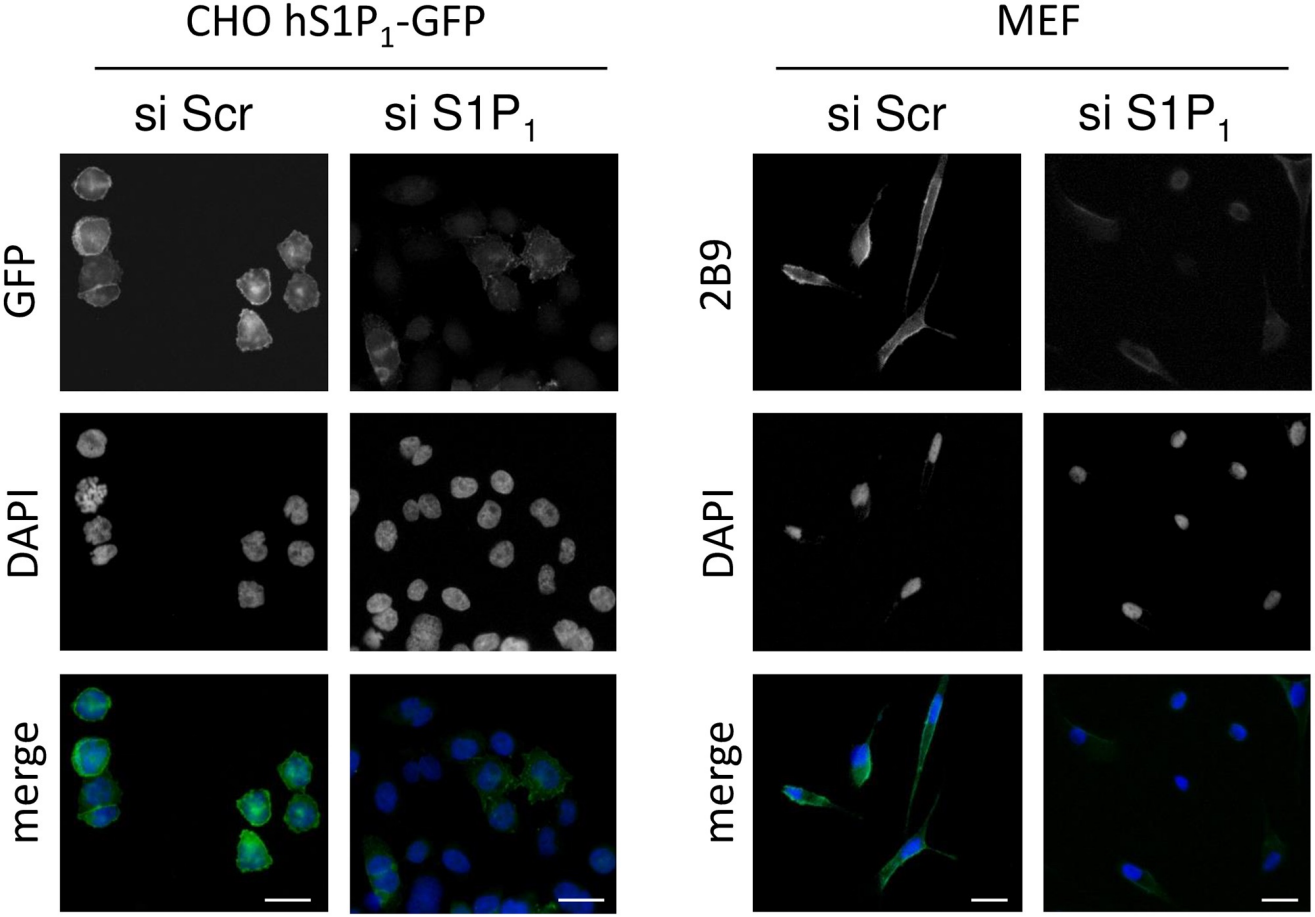
S1P_1_ knockdown leads to loss of 2B9 staining. CHO cells stably expressing hS1P_1_-GFP (CHO hS1PR1-GFP) and MEF were transfected with siRNA against S1P_1_ (si S1P_1_) or scramble (si Scr). At 48 hours post transfection, CHO cells were fixed and stained for DNA with DAPI (blue). MEFs were fixed and stained for S1P_1_ with 2B9 (1:100, w/v, green) and DNA with DAPI (blue). Images are representative of the population examined. Scale bar, 20 μm.

We next characterized 2B9 by immunocytochemistry. Fig 4 shows that 2B9 recognizes S1P_1_-GFP protein since 2B9 colocalized with GFP signal at the level of the plasma membrane. An IgG2A control antibody did not stain CHO S1P_1_-GFP cells suggesting the specificity of 2B9. Importantly, 2B9 stained plasma membrane similarly to the widely used commercial H60 antibody [11, 25]. Neither 2B9 nor IgG2A stained CHO-GFP cells further confirming that 2B9 interacts with S1P_1_ and the GFP tag did not affect this interaction. Lastly, in agreement with the literature, no staining was observed in WT CHO cells transfected with pcDNA3 plasmid that do not express any S1P receptor [24] (Fig 4). The epitope detected by 2B9 is localized to the intracellular domains of S1P_1_ since strong membrane staining was observed in permeabilized cells while 2B9 did not stain non-permeabilized cells (Fig 5). We investigated whether 2B9 recognizes untagged human and mouse S1P_1_. To answer this question, receptors were stably expressed in CHO cells (CHO hS1P_1_; CHO mS1P_1_). 2B9 specifically stained hS1P_1_ at the level of plasma membrane while no staining was observed with control IgG2A antibodies. Furthermore, specific membrane staining was observed in cells expressing mouse S1P_1_ (Fig 6). These results demonstrate that 2B9 interacts with both human and mouse S1P_1_ receptors. A major challenge in S1P receptor studies is the detection of endogenous receptors. We have previously shown that S1P_1_ mRNA is present in primary MEF suggesting that protein may be also expressed [17]. Since 2B9 recognizes mouse S1P_1_, we asked whether it was able to interact with the endogenous mS1P_1_. As shown in Fig 7, 2B9 revealed punctate cytoplasmic as well as membrane (arrows and enlarged merged image) S1P_1_ localization while no staining was observed with the control IgG2A antibody. We next examined whether 2B9 recognized human endogenous S1P_1_. Cytoplasmic as well as plasma membrane S1P_1_ localization was observed with 2B9 in the BT-549 breast cancer cell line (Fig 7). Taken together our results indicate that 2B9 is able to reveal endogenous human and mouse S1P_1_. To further confirming the specificity of 2B9, we finally tested whether knockdown of the S1P_1_ by specific S1P_1_ small interfering RNA (si RNA) as previously described [19] leads to loss of S1P_1_ staining. First, CHO S1P_1_-GFP cells were transfected with control (scramble, siScr) or si RNAs against S1P_1_ (siS1P_1_) and immunocytochemistry was performed 48 hours later. As shown in Fig 8, siS1P_1_ treatment completely abolished receptor staining as compared to control cells (siScr) demonstrating the efficacy of the S1P_1_ siRNAs. Second, to further validating the specificity of 2B9 we tested whether S1P_1_ knockdown could trigger loss of endogenous S1P_1_ staining. Membrane S1P_1_ labeling in MEF was indeed completely abrogated in cells treated with siS1P_1_ as compared to control cells (Fig 8). These data thus establish that 2B9 specifically recognizes endogenous S1P_1_.

**Fig 9:**
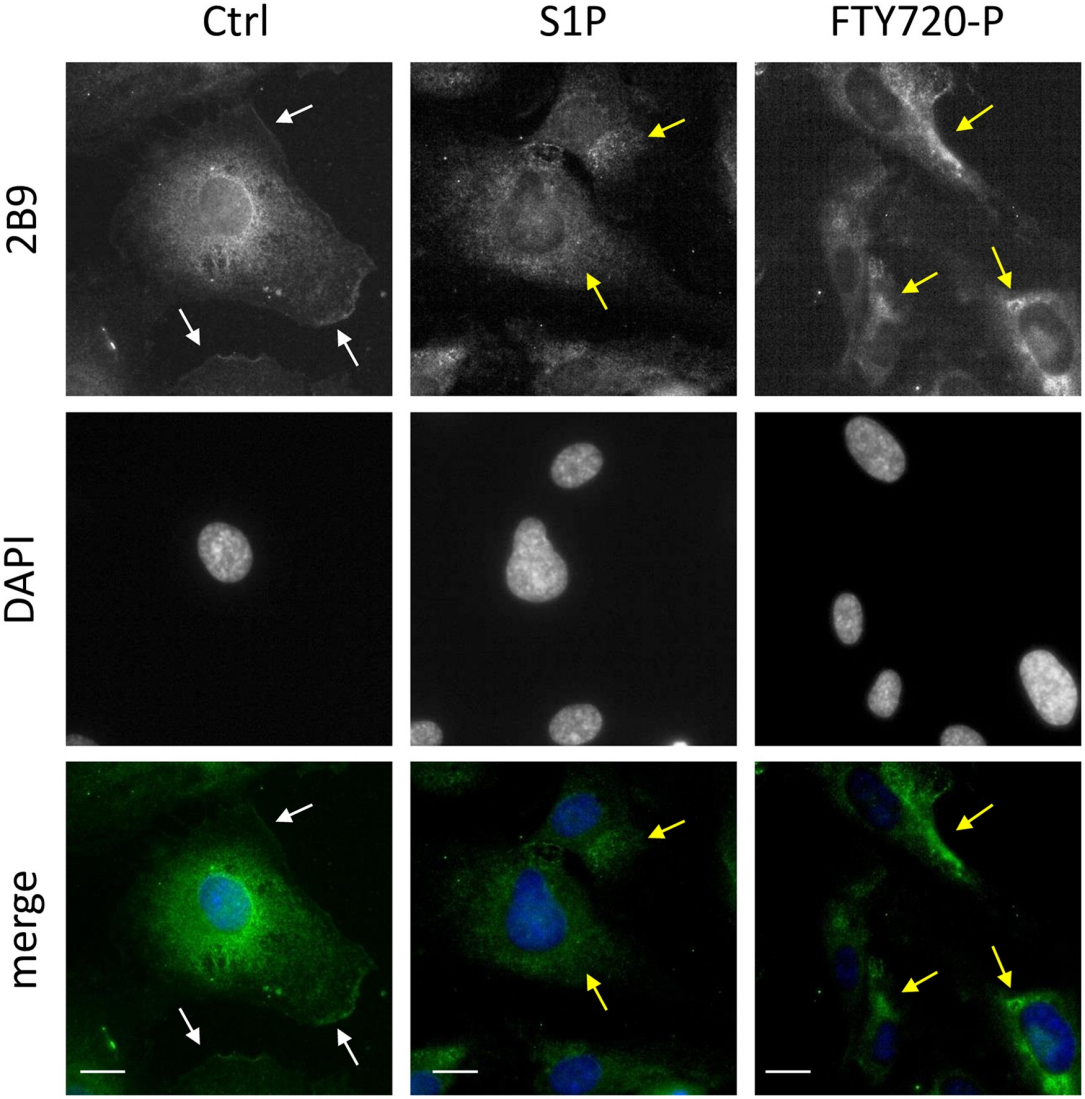
Endogenous S1P_1_ agonist-induced internalization can be followed with 2B9. HUVEC cells were seeded on coverslips and 24h later were serum starved for 2 hours. Cells were then stimulated with either vehicle (Ctrl), S1P (1μM) or FTY720-P (1μM) for 30 min, then fixed, permeabilized and stained with 2B9 (1:100, w/v, green) and DAPI (blue). Under serum starvation conditions (Ctrl) S1P_1_ was localized at the plasma membrane and cytoplasm. In the presence of S1P or FTY720-P, S1P_1_ is localized within cytoplasm. White arrows indicate S1P_1_ staining at the level of plasma membrane in non-treated cells and yellow arrows intracellular staining in S1P and FTY720-P treated cells. Images are representative of the population examined. Scale bar, 10 μm.

S1P_1_ receptor undergoes rapid internalization upon agonist stimulation [9, 27]. We next followed endocytosis of endogenous S1P_1_ in HUVEC cells using 2B9. As shown in Fig 9, under serum starvation conditions, 2B9 detected cytoplasmic as well as membrane S1P_1_ in control cells (Ctrl). White arrows indicate that part of S1P_1_ localized at the level of the plasma membrane. Treatment with S1P or FTY720-P rapidly induced receptor endocytosis and internalization since S1P_1_ is localized only within the cytoplasm (yellow arrows). Our data confirm that pharmacological agonists induce receptor internalization and that 2B9 is well suited to study endogenous S1P_1_ endocytosis.

### 2B9 detects S1P_1_ in human spinal cord and kidney by immunohistochemistry

**Fig 10:**
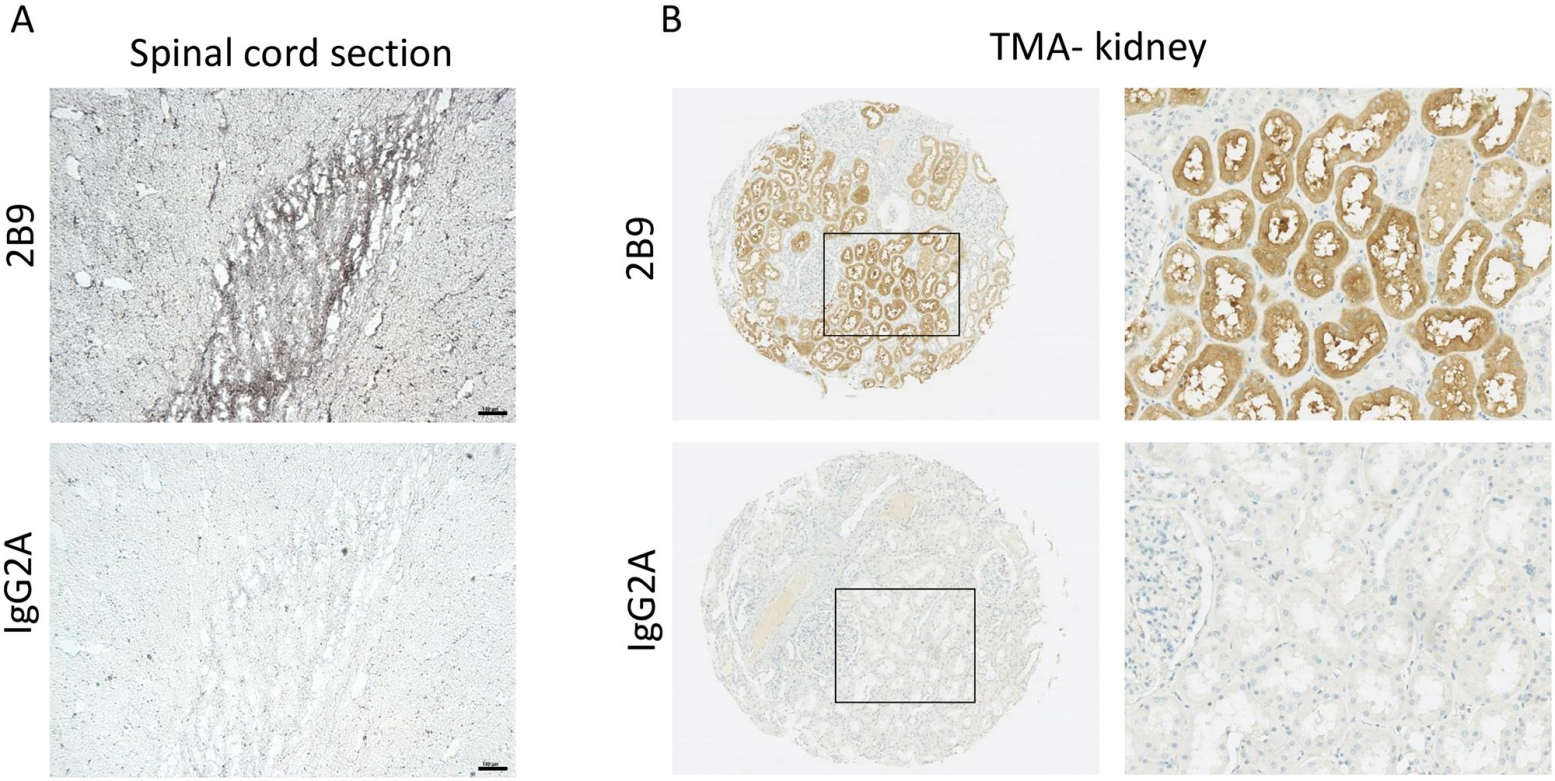
S1P_1_ is expressed in human tissues. S1P_1_ is expressed in human spinal cord. Immunohistochemistry was performed in frozen human spinal cord sections using 2B9 and control IgG2A antibodies as described in methods section. Scale bar, 100 μm. A, S1P_1_ is expressed in human kidney. Immunohistochemistry was performed in human TMA kidney samples using 2B9 and control IgG2A antibodies (1:50, w/v) as was previously described [23]. Right panel shows 2mm TMA kidney sections, left panel shows X20 magnification.

Because expression of S1P_1-5_ is often dysregulated in pathological conditions, detection of S1P_1_ at tissue level is of crucial importance. Firstly, immunohistochemistry (IHC) on frozen human spinal cord sections was conducted as the presence of S1P_1_ in human spinal cord section was previously reported using H60 polyclonal antibodies [28]. Fig 10A, revealed the specific 2B9 staining at tissue level as compared to control IgG2A antibodies. Secondly, we showed the specific S1P_1_ staining in the epithelial cells of the collecting duct of human kidney (Fig 10B) using 2B9 antibodies, as compared to control IgG2A, in line with other studies [25, 29].

## Discussion

S1P_1-5_ are differentially expressed under various physiological and pathological conditions [30, 31]. Despite the plethora of studies describing the cellular functions of S1P receptors, tools that allow detection, quantification and localization of these receptors are unfortunately scarce. In fact, most of the currently available antibodies directed at GPCRs are not correctly validated neither for specificity nor for the detection of endogenously expressed receptors [16, 32, 33]. A challenge for GPCR receptor studies and more particularly S1P receptors studies is the specific and accurate detection of endogenous proteins. To this purpose, we developed and characterized a new monoclonal anti-S1P_1_ antibody, named 2B9. Based on our previous work [14, 15, 34], we expressed and purified the human S1P_1_ in the *Pichia pastoris* model, which allows a large availability of pure proteins. Immunization of mice with a SDS-solubilized S1P_1_ induced an immune reaction against foreign antigens and nine hybridoma cell lines producing antibodies were established. The advantage of the hybridoma model is the unlimited production of the same monoclonal antibody while polyclonal antibody production is animal-dependent. This is the case for the commonly used rabbit anti-S1P_1_ antibody sc-25489 (H60) from Santa Cruz Biotechnology Company. This antibody was used in a great number of S1P_1_ studies [9, 13, 25, 26, 28, 35–42] but is not anymore commercially available. In this work, we show that 2B9 specifically binds to S1P_1_ expressed either as endogenous or recombinant in various cell lines. 2B9 detected, by Western blot, recombinant mouse and human S1P_1_ receptors expressed in CHO and HEK cells as well as the endogenous human and mouse receptors present in cell lines of different origin, like primary cells (HUVEC and MEF) and cancer derived cells (BT-549). Variation in the molecular weight of S1P_1_ among cell lines can be explained at least in part by posttranslational modification such as glycosylation [43–45]. S1P_1_ exhibit one potential glycosylation site located at position 30 (asparagine) in the amino-terminus extra-cellular part of the receptor. Asparagine 30 N-glycosylation was formally detected in recombinant HEK cells [44] but not in recombinant *Pichia pastoris*. In general, proteins expressed in *P*. *pastoris* have shorter glycosylation chains than those expressed in *Saccharomyces cerevisiae* thus making *P*. *pastoris* a more attractive host for the expression of recombinant proteins.

Some 2B9 binds to the cmyc-hS1P_1_ in HEK cells, but does not interact with cmyc-hS1P_2_ and cmyc-hS1P_5_ suggesting its specificity. Finally, endogenous receptors in HUVEC and MEF cells were detected, after immunoprecipitation, as a large protein band, in accordance with previous studies [46]. Our results show that 2B9 is suitable for immunoprecipitation. This potential is essential in the context of cell or tissue analyses where variation of very low expression levels correlate with physiopathological conditions. Specific and dynamic subcellular localization of S1P_1_ has been correlated to its cellular functions. S1P_1_ cytoplasmic, membrane, as well as nuclear localization have been reported in recombinant [47–50] and endogenous cells [38, 42, 51]. Furthermore, subcellular S1P receptors localization reflects the pharmacological action of receptor specific agonists and antagonists [27]. Thus developing antibodies appropriate for cell imaging studies contributes to the understanding of S1P receptors trafficking at the cellular level. In agreement with previous studies that used the CHO hS1P_1_-GFP cell line, we showed that 2B9 detects hS1P_1_ at the plasma membrane [52]. When expressed in other cell lines [47–50], hS1P_1_-GFP was also found at the plasma membrane. More importantly, we showed cytoplasmic and membrane staining of the endogenous human and mouse receptors using 2B9 in various cells (HUVEC, MEF and BT-549 breast cancer cell line). This labeling is correlate well with specific concern since the control IgG2A antibody did not show any staining and knockdown of the S1P_1_ receptor with specific siRNAs [17] led to the loss of immunofluorescent signal. Taken together, our results demonstrate that 2B9 is suitable for endogenous S1P_1_ immunocytochemistry studies. Immunofluorescence performed on intact and permeabilized cells demonstrated that 2B9 recognizes an epitope located at the intracellular part of S1P_1_. Thus, various glycosylation patterns encountered at different cells will have no impact on the detection of S1P_1_. S1P receptors are considered as therapeutic targets and more particularly S1P_1_ in multiple sclerosis. The oral sphingosine-1-phosphate receptor modulator Fingolimod (FTY720) was described to induce receptor endocytosis and to prevent lymphocyte egress from lymphoid tissues [53]. For this reason, we investigated whether we can study ligand-induced receptor internalization using 2B9. Indeed, in agreement with the literature [9, 11, 47, 48, 50, 51, 54–56] treatment of cells with S1P or FTY720-P led to the internalization of the plasma membrane S1P_1_ receptor suggesting that 2B9 is suitable for receptor endocytosis studies upon ligand stimulation.

The detection of the S1P_1_ receptor by immunohistochemistry is important in tissue since its expression is related to physiopathology [1, 10, 53, 57]. 2B9 antibody detected S1P_1_ in human adult kidney and spinal cord in agreement with previous studies [25, 28, 29] suggesting that 2B9 is appropriate for immunohistochemistry studies. The current knowledge of therapeutic potentials of S1P_1_ in disorders including inflammation, fibrosis, and cancer underlines the importance of tools that allow the monitoring of deregulated S1P_1_ expression. In conclusion, our work participates to the production and characterization of a new tool for the detection, localization and quantitation of endogenous S1P_1_ receptor expression. We must finally advise researchers that all results obtained with an antibody on recombinant cells or on tissues or endogenous cells have to be compared with results obtained with a control.

## Supporting information

S1 File Arrive guideline checklist. (PDF)
